# Adverse events as potential predictive factors of therapeutic activity in patients with unresectable hepatocellular carcinoma treated with atezolizumab plus bevacizumab

**DOI:** 10.1002/cam4.5535

**Published:** 2022-12-14

**Authors:** Toshifumi Tada, Takashi Kumada, Atsushi Hiraoka, Masashi Hirooka, Kazuya Kariyama, Joji Tani, Masanori Atsukawa, Koichi Takaguchi, Ei Itobayashi, Shinya Fukunishi, Kunihiko Tsuji, Toru Ishikawa, Kazuto Tajiri, Hironori Ochi, Satoshi Yasuda, Hidenori Toyoda, Chikara Ogawa, Takashi Nishimura, Takeshi Hatanaka, Satoru Kakizaki, Noritomo Shimada, Kazuhito Kawata, Fujimasa Tada, Hideko Ohama, Kazuhiro Nouso, Asahiro Morishita, Akemi Tsutsui, Takuya Nagano, Norio Itokawa, Tomomi Okubo, Taeang Arai, Michitaka Imai, Hisashi Kosaka, Atsushi Naganuma, Yohei Koizumi, Shinichiro Nakamura, Masaki Kaibori, Hiroko Iijima, Yoichi Hiasa

**Affiliations:** ^1^ Division of Gastroenterology and Hepatology, Department of Internal Medicine Hyogo Medical University Nishinomiya Japan; ^2^ Department of Internal Medicine Japanese Red Cross Himeji Hospital Himeji Japan; ^3^ Department of Nursing Gifu Kyoritsu University Ogaki Japan; ^4^ Gastroenterology Center Ehime Prefectural Central Hospital Matsuyama Japan; ^5^ Department of Gastroenterology and Metabology Ehime University Graduate School of Medicine Ehime Japan; ^6^ Department of Gastroenterology Okayama City Hospital Okayama Japan; ^7^ Department of Gastroenterology and Hepatology Kagawa University Kagawa Japan; ^8^ Division of Gastroenterology and Hepatology, Department of Internal Medicine Nippon Medical School Tokyo Japan; ^9^ Department of Hepatology Kagawa Prefectural Central Hospital Takamatsu Japan; ^10^ Department of Gastroenterology Asahi General Hospital Asahi Japan; ^11^ Department of Gastroenterology Osaka Medical and Pharmaceutical University Osaka Japan; ^12^ Center of Gastroenterology Teine Keijinkai Hospital Sapporo Japan; ^13^ Department of Gastroenterology Saiseikai Niigata Hospital Niigata Japan; ^14^ Department of Gastroenterology Toyama University Hospital Toyama Japan; ^15^ Hepato‐biliary Center Japanese Red Cross Matsuyama Hospital Matsuyama Japan; ^16^ Department of Gastroenterology and Hepatology Ogaki Municipal Hospital Ogaki Japan; ^17^ Department of Gastroenterology Japanese Red Cross Takamatsu Hospital Takamatsu Japan; ^18^ Department of Gastroenterology Gunma Saiseikai Maebashi Hospital Maebashi Japan; ^19^ Department of Clinical Research National Hospital Organization Takasaki General Medical Center Takasaki Japan; ^20^ Division of Gastroenterology and Hepatology Otakanomori Hospital Kashiwa Japan; ^21^ Department of Hepatology Hamamatsu University School of Medicine Hamamatsu Japan; ^22^ Department of Surgery Kansai Medical University Osaka Japan; ^23^ Department of Gastroenterology National Hospital Organization Takasaki General Medical Center Takasaki Japan

**Keywords:** adverse event, atezolizumab plus bevacizumab, hepatocellular carcinoma, survival

## Abstract

**Aim:**

To investigate the possible correlation between the development of adverse events (AEs) and prognosis in patients with unresectable hepatocellular carcinoma (HCC) treated with atezolizumab plus bevacizumab (Atez/Bev).

**Methods:**

A total of 286 patients with unresectable HCC treated with Atez/Bev as first‐line systematic therapy were included.

**Results:**

Regarding treatment‐related AEs, decreased appetite of any grade, proteinuria of any grade, and fatigue of any grade were found with a frequency of ≥20%. Multivariate analysis adjusted for immune‐related liver injury, immune‐related endocrine dysfunction, proteinuria, fatigue, decreased appetite, hypertension, sex, age, Eastern Cooperative Oncology Group performance status, HCC etiology, HCC stage, Child–Pugh score, and α‐fetoprotein showed that hypertension of any grade (hazard ratio [HR], 0.527; 95% confidence interval [CI], 0.326–0.854; *p* = 0.009) and α‐fetoprotein ≥100 ng/ml (HR, 1.642; 95% CI, 1.111–2.427; *p* = 0.013) were independently associated with progression‐free survival. Multivariate analysis adjusted for the same AEs showed that fatigue (HR, 2.354; 95% CI, 1.299–4.510; *p* = 0.010) was independently associated with overall survival. Median progression‐free survival was 6.5 months (95% CI, 5.2–8.1) in patients without hypertension of any grade and 12.6 months (95% CI, 6.7–not available) in patients with hypertension of any grade (*p* = 0.035). The overall survival was significantly shorter in patients in whom treatment‐related fatigue of any grade was observed (*p* < 0.001). Regarding response rates, the disease control rate of patients who developed treatment‐related hypertension (94.2%) was significantly higher than those who did not (79.1%) (*p* = 0.009).

**Conclusions:**

Treatment‐related hypertension is associated with good outcomes in patients with HCC treated with Atez/Bev.

## Graphical abstract

Treatment‐related decreased appetite, proteinuria, and fatigue were found with a frequency of =20% during atezolizumab plus bevacizumab therapy for hepatocellular carcinoma. Treatment‐related hypertension was associated with good outcomes in patients treated with atezolizumab plus bevacizumab. Treatment‐related fatigue was associated with poor outcome in patients treated with atezolizumab plus bevacizumab.

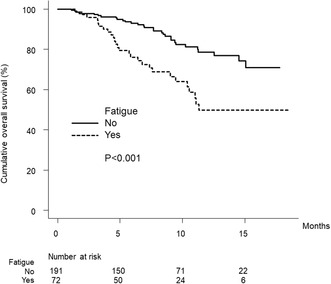

## 1 INTRODUCTION

Primary liver cancer was the 6th most frequently diagnosed neoplasm and the 3rd major cause of cancer‐related mortality worldwide in 2020, with approximately 906,000 new cases diagnosed and 830,000 deaths.^1^ Hepatocellular carcinoma (HCC) comprises approximately 75%–85% of primary liver cancers^1^ and constitutes a leading health issue worldwide. Curative treatments such as hepatectomy, transplantation, and percutaneous local puncture ablation therapy are performed for patients with Barcelona Clinic Liver Cancer (BCLC) early‐stage HCC.^2^ Majority of patients who are not candidates for curative surgical or ablative treatment receive transarterial chemoembolization, radiation therapy, or systemic drug therapy for palliative treatment.^2^

Atezolizumab plus bevacizumab (Atez/Bev) was recently developed and confirmed as 1st‐line systemic therapy for patients with unresectable HCC. This 1st‐line systemic therapy is comprised of the combination of an immune checkpoint inhibitor (Atez) and a molecular targeted agent (Bev).^3^ It is found to have stronger therapeutic impact, including improved outcomes in HCC patients, than previously used 1st‐line systemic therapies such as sorafenib and lenvatinib.^3^, ^4^

Management of adverse events (AEs) is important to prolong survival of patients with malignant disease receiving systemic therapy. Several studies have investigated the relationship between the AEs development in patients who were treated with immune checkpoint inhibitors or molecular targeted agents and their outcomes in various malignancies.^5^, ^6^, ^7^, ^8^, ^9^, ^10^ In patients with HCC, a relationship between the occurrence of certain AEs and prognosis has been reported in patients who were treated with tyrosine kinase inhibitors such as sorafenib and lenvatinib.^11^, ^12^, ^13^ Our multicenter study group has recently published clinical data including AEs in HCC patients treated with Atez/Bev.^14^, ^15^, ^16^, ^17^, ^18^, ^19^, ^20^, ^21^ However, the relationship between occurrence of AEs and outcomes in patients who were treated with Atez/Bev for BCLC intermediate and advanced stage HCC has not been adequately researched in the real clinical practice.

With this aim, we performed a large‐scale retrospective investigation in patients with HCC who were treated with Atez/Bev in a 1st‐line systemic therapy setting at multicenter of liver disease in Japan, and researched the possible relationship between the development of AEs and three different outcome assessment: overall survival, progression‐free survival, and therapeutic response.

## 2 MATERIALS AND METHODS

### 2.1 Patients

This multicenter study was implemented as a retrospective database research according to the Guidelines for Clinical Research created by the Ministry of Health and Welfare of Japan after receiving the Ministry's official approval. This retrospective research protocol was approved by the Institutional Ethics Committee of Ehime Prefectural Central Hospital (IRB no. 30–66) (UMIN000043219). All process of this study were performed based on the Declaration of Helsinki. In this study, written informed consent was obtained from all patients who participated in this study.

Between September 2020 and March 2022, a total of 467 patients with unresectable HCC were treated with Atez/Bev at 22 institutions in Japan. Of these, 263 patients who met the following eligibility criteria were enrolled: (1) Atez/Bev was used for HCC as the 1st‐line systemic therapy, and (2) clinical data including therapeutic response were available.

The HCC etiology was defined to be hepatitis C virus in patients with positive for hepatitis C virus antibodies, and hepatitis B virus in patients with positive for hepatitis B virus surface antigen.

The date of initiation of Atez/Bev therapy was determined as the start of follow‐up. The end of follow‐up was determined as the date of the last visit for patients who survived and the date of death for patients who died during the observation period.

### 2.2 Confirmation and treatment of HCC

In this study, HCC was confirmed based on one or more of the following: increased α‐fetoprotein levels, typical imaging findings on gadolinium ethoxybenzyl diethylenetriamine pentaacetic acid–enhanced magnetic resonance imaging, dynamic‐computed tomography (CT), or contrast‐enhanced ultrasonography, and pathological findings.^22^, ^23^ HCC stage was determined according to the BCLC classification system.^24^

The optimal treatment for HCC for each patient was determined by discussions among hepatologists, surgeons, oncologists, and radiologists at each hospital in accordance with Japanese practice guidelines for HCC.^25^, ^26^

### 2.3 Atez/Bev treatment and AE evaluation

After affirmation written informed consent from each patient, intravenous Atez/Bev therapy comprising of 1200 mg of Atez plus 15 mg/kg of body weight of Bev was given every 3 weeks.^3^ This Atez/Bev therapy was stopped if clinical tumor progression or any serious or unacceptable AEs occurred during this treatment.

AEs were evaluated using the National Cancer Institute Common Terminology Criteria for Adverse Events, version 5.0.^27^ In the present study, treatment‐related AEs, including those that were immune related, were diagnosed by the attending physician. If an AE developed, the guidelines for Atez/Bev therapy created by the manufacturer of these drugs were used to determine if either or both drugs should be reduced in dose or discontinued. If Atez/Bev therapy was stopped, attending physician at each hospital made decisions about introducing another treatment according to Japanese practice guidelines for HCC.^25^, ^26^

### 2.4 Evaluation of therapeutic response

The Response Evaluation Criteria in Solid Tumors, ver. 1.1,^28^ was adopted to evaluate radiological therapeutic response [complete response (CR), partial response (PR), stable disease (SD), progressive disease (PD)]. Whenever possible, initial evaluation of the therapeutic effect was carried out using dynamic CT findings obtained 6 weeks after the introduction of Atez/Bev, then additional dynamic CT examinations were carried out as needed depending on the patient's condition, sometimes even within 6 weeks after the initial evaluation. Beyond 6 weeks, dynamic CT assessment for therapeutic response were carried out every 6 weeks and then every 9–12 weeks after the first 6 months.

The best responses were defined after excluding patients who did not undergo imaging assessment due to a short follow‐up period (i.e., the final observation date was prior to the imaging assessment).

### 2.5 Statistical analysis

Continuous variables are described as medians (interquartile range). The progression‐free survival was determined as the duration between the date of Atez/Bev started and the date of PD or death, and the overall survival was determined as the duration between the date of Atez/Bev started and the end of follow‐up. Evaluation of cumulative progression‐free survival and overall survival was carried out using the Kaplan–Meier method, and statistical differences were evaluated with the log‐rank test. Progression‐free (events, *n* = 131) and overall (events, *n* = 57) survival and their hazard ratios (HRs) were determined by multivariate Cox proportional hazards modeling that adjusted for AEs of any grade that occurred with a frequency of ≥10% in the study population. In the multivariate Cox proportional hazards modeling for progression‐free survival, we additionally adjusted the following factors that were previously reported to be predictors of liver disease prognosis or risk factors for HCC: sex, age, HCC etiology, Eastern Cooperative Oncology Group performance status (ECOG‐PS), HCC stage, α‐fetoprotein, and Child–Pugh score.^29^, ^30^ In the present study, we used the following cut‐off levels of continuous clinical factors for assessment: age, 75 years and α‐fetoprotein, 100 ng/ml according to previous reports.^29^, ^30^

Statistical difference was defined as *p* < 0.05. Statistical analyses were carried out with EZR version 1.55 (Saitama Medical Center, Jichi Medical University), which is a graphical user interface for R (The R Foundation for Statistical Computing).^31^

## 3 RESULTS

### 3.1 Characteristics of study patient

The characteristics of the 263 patients are listed in Table 1. There were 55 (20.9%) females and 208 (79.1%) males, with a median age of 74.0 years (68.0–79.0). The median observation period was 8.3 months (5.2–11.8). No patients in this cohort received the combination of Atez/Bev and locoregional therapies such as transarterial chemoembolization.

**TABLE 1:** Patient characteristics (*n* = 263)

Age^a^ (years)	74.0 (68.0–79.0)
Sex (Female/Male)	55/208
ECOG‐PS (0/1/≥2)	216/35/11
Body mass index (kg/m^2^)	23.7 (21.2–26.1)
Etiology of HCC (hepatitis B/C/B + C/non‐B, non‐C)	41/94/1/127
Albumin (g/dl)^a^	3.8 (3.4–4.1)
Total bilirubin (mg/dl)^a^	0.8 (0.6–1.1)
Platelet count (×10^3^/m^3^)^a^	13.7 (10.3–19.8)
Prothrombin time (%)^a^	88 (80–97)
α‐fetoprotein level (ng/ml)^a^	31.4 (6.5–298.8)
Child–Pugh score (5/6/≥7)	163/80/20
Tumor size (cm)^a^	3.1 (1.8–7.0)
Number of tumors in the liver (0/1/2/3/4/≥5)	16/61/34/23/24/128
BCLC stage (≤A/B/≥C)	21/100/142
Follow‐up duration^a^ (months)	8.3 (5.2–11.8)

Abbreviations: BCLC, Barcelona Clinic Liver Cancer; ECOG‐PS, Eastern Cooperative Oncology Group Performance Status; HCC, hepatocellular carcinoma.

^a^ Data expressed as medians (interquartile range).

### 3.2 Progression‐free and overall survival

**FIGURE 1:**
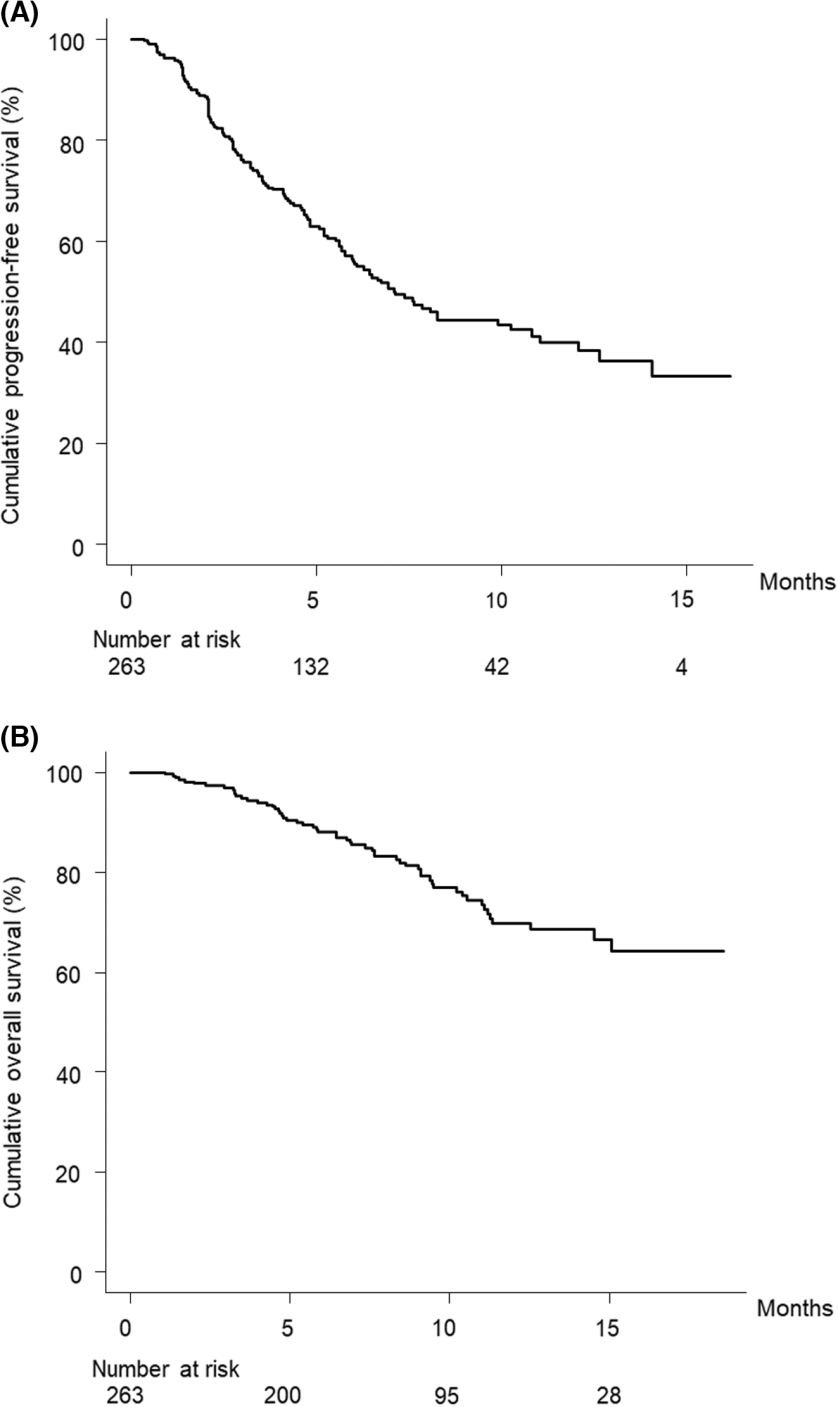
(A) Cumulative progression‐free survival curve. The cumulative progression‐free survival rates at 3, 6, and 12 months are 76.1%, 56.1%, and 40.1%, respectively. (B) Cumulative overall survival curve. The cumulative overall survival rates at 3, 6, and 12 months are 96.9%, 88.1%, and 69.9%, respectively.

Figure 1A shows the curve for progression‐free survival. The median progression‐free survival time was 7.1 months (95% confidence interval [CI], 6.0–10.2).

Figure 1B shows the curve for overall survival. The median overall survival time was not available (NA) months (95% CI, NA–NA).

### 3.3 Therapeutic response

The radiological best response rates for CR, PR, SD, and PD were 4.9%, 27.0%, 50.2%, and 17.9%, respectively (Table 2). The overall response rate (ORR) was 31.9%, and the disease control rate (DCR) was 82.1% (Table 2).

**TABLE 2:** Therapeutic response (*n* = 263)

CR	13 (4.9%)
PR	71 (27.0%)
SD	132 (50.2%)
PD	47 (17.9%)
ORR	31.9%
DCR	82.1%

Abbreviations: CR, complete response; DCR; disease control rate; ORR, overall response rate; PD, progressive disease; PR, partial response; SD, stable disease.

### 3.4 AEs

Table 3 lists the treatment‐related AEs in this study. Decreased appetite of any grade, proteinuria of any grade, and fatigue of any grade all occurred at a frequency of ≥20%. In this study, there were no patients with treatment‐related hypertension of grade 4.

**TABLE 3:** AEs

Immune‐related liver injury	
Any grade	30 (11.4%)
Grade ≥3	5 (1.9%)
Immune‐related endocrine dysfunction	
Any grade	29 (11.0%)
Grade ≥3	3 (1.1%)
Immune‐related other	
Any grade	48 (18.3%)
Grade ≥3	6 (2.3%)
Proteinuria	
Any grade	87 (33.1%)
Grade ≥3	24 (9.1%)
Fatigue	
Any grade	72 (27.4%)
Grade ≥3	7 (2.7%)
Decreased appetite	
Any grade	61 (23.2%)
Grade ≥ 3	6 (2.3%)
Hypertension	
Any grade	52 (19.8%)
Grade ≥ 3	13 (4.9%)
Other	
Any grade	107 (40.7%)
Grade ≥ 3	36 (13.7%)

Abbreviations: AE, adverse event.

### 3.5 AEs associated with progression‐free and overall survival

Multivariate analysis showed that hypertension of any grade (HR, 0.527; 95% CI, 0.326–0.854; *p* = 0.009) and α‐fetoprotein ≥100 ng/ml (HR, 1.642; 95% CI, 1.111–2.427; *p* = 0.013) were independently related to progression‐free survival (Table 4), while fatigue of any grade (HR, 2.354; 95% CI, 1.299–4.510; *p* = 0.010) was independently related to overall survival (Table 5).

**TABLE 4:** Multivariate analysis of progression‐free survival

	HR	95% CI	*p* value
Immune‐related liver injury			
No (*n* = 233)	1	0.827–2.503	0.198
Yes (*n* = 30)	1.439		
Immune‐related endocrine dysfunction			
No (*n* = 234)	1	0.335–1.157	0.134
Yes (*n* = 29)	0.623		
Proteinuria			
No (*n* = 176)	1	0.457–1.009	0.056
Yes (*n* = 87)	0.679		
Fatigue			
No (*n* = 191)	1	0.716–1.768	0.610
Yes (*n* = 72)	1.125		
Decreased appetite			
No (*n* = 202)	1	0.398–2.490	0.089
Yes (*n* = 61)	1.528		
Hypertension			
No (*n* = 211)	1	0.326–0.854	0.009
Yes (*n* = 52)	0.527		
Age (years)			
<75 (*n* = 144)	1	0.650–1.346	0.720
≥75 (*n* = 119)	0.936		
Sex			
Female (*n* = 55)	1	0.813–1.959	0.299
Male (*n* = 208)	1.262		
ECOG‐PS			
0 (*n* = 216)	1	0.787–2.208	0.294
≥1 (*n* = 47)	1.318		
Etiology			
Viral (*n* = 136)	1	0.704–1.452	0.951
Non‐viral (*n* = 127)	1.011		
α‐fetoprotein (ng/ml)			
<100 (*n* = 170)	1	1.111–2.427	0.013
≥100 (*n* = 93)	1.642		
BCLC stage			
≤B (*n* = 121)	1	0.698–1.580	0.814
≥C (*n* = 142)	1.050		
Child–Pugh score			
≤6 (*n* = 243)	1	0.785–2.732	0.231
≥7 (*n* = 20)	1.464		

Abbreviations: BCLC, Barcelona Clinic Liver Cancer; CI, confidence interval; ECOG‐PS, Eastern Cooperative Oncology Group Performance Status; HR, hazard ratio.

**TABLE 5:** Multivariate analysis of overall survival

	HR	95% CI	*p* value
Immune‐related liver injury			
No (*n* = 233)	1	0.720–3.053	0.285
Yes (*n* = 30)	1.483		
Immune‐related endocrine dysfunction			
No (*n* = 234)	1	0.466–2.111	0.982
Yes (*n* = 29)	0.992		
Proteinuria			
No (*n* = 176)	1	0.457–1.419	0.453
Yes (*n* = 87)	0.805		
Fatigue			
No (*n* = 191)	1	1.229–4.510	0.010
Yes (*n* = 72)	2.354		
Decreased appetite			
No (*n* = 202)	1	0.654–2.505	0.472
Yes (*n* = 61)	1.280		
Hypertension			
No (*n* = 211)	1	0.393–1.457	0.405
Yes (*n* = 52)	0.757		

Abbreviations: CI, confidence interval; HR, hazard ratio.

### 3.6 Progression‐free and overall survival according to statistically significant AEs

**FIGURE 2:**
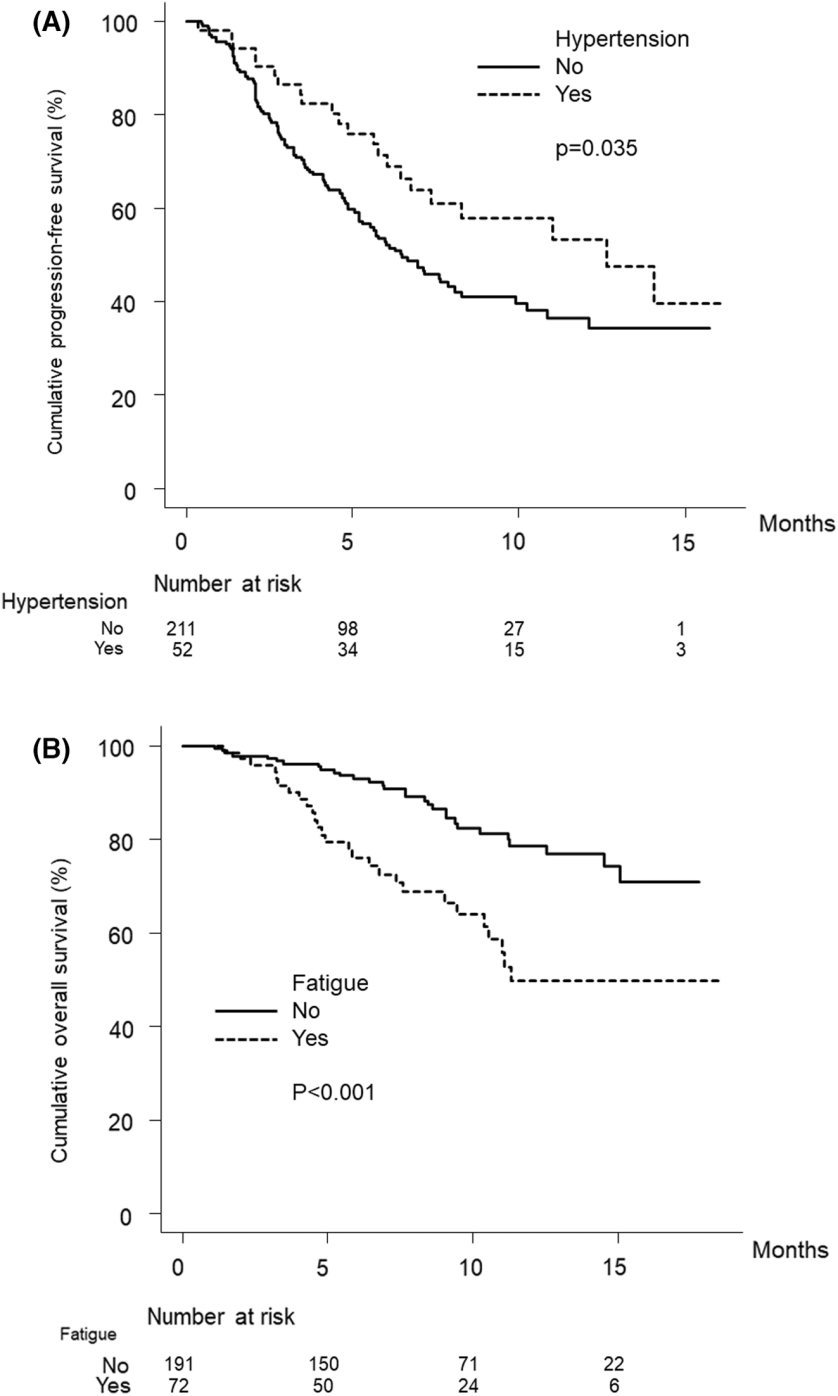
(A) Cumulative progression‐free survival curves with or without treatment‐related hypertension. The cumulative progression‐free survival rates of patients with hypertension at 3, 6, and 12 months are 86.5%, 71.3%, and 53.3%, respectively (dotted line). In patients without hypertension, the cumulative progression‐free survival rates are 73.5%, 52.1%, and 36.4% at 3, 6, and 12 months, respectively (solid line) (*p* = 0.035, log‐rank test). (B) Cumulative overall survival curves with or without treatment‐related fatigue. The cumulative overall survival rates of patients with fatigue at 3, 6, and 12 months are 95.8%, 76.1%, and 49.9%, respectively (dotted line). In patients without fatigue, the cumulative overall survival rates are 97.3%, 93.0%, and 78.5% at 3, 6, and 12 months, respectively (solid line) (*p* < 0.001, log‐rank test).

Figure 2A shows the curves for progression‐free survival stratified by the presence or absence of treatment‐related hypertension of any grade. The median progression‐free survival was 6.5 months (95% CI, 5.2–8.1) and 12.6 months (95% CI, 6.7–NA) in patients without or with hypertension of any grade, respectively (*p* = 0.035).

Figure 2B shows the curves for overall survival stratified by the presence or absence of treatment‐related fatigue of any grade. The median overall survival was NA months (95% CI, NA–NA) and 11.3 months (95% CI, 10.2–NA) in patients without or with fatigue of any grade, respectively. The overall survival was significantly shorter in patients in whom treatment‐related fatigue of any grade was observed (*p* < 0.001). The baseline characteristics of the study patients stratified by the presence or absence of treatment‐related fatigue of any grade are summarized in Table S1.

### 3.7 Therapeutic response rates stratified by the presence or absence of AEs

The radiological best response rates were significantly different when stratified by the presence or absence of treatment‐related hypertension of any grade (*p* = 0.042) (Table 6). The DCR was significantly different between patients with and without treatment‐related hypertension of any grade (*p* = 0.009) (Table 6).

**TABLE 6:** Therapeutic response by treatment‐related hypertension of any grade

	Yes (*n* = 52)	No (*n* = 211)	*p* value
CR	3 (5.8%)	10 (4.7%)	0.042
PR	18 (34.6%)	53 (25.1%)	
SD	28 (53.8%)	104 (49.3%)	
PD	3 (5.8%)	44 (20.9%)	
ORR	40.4%	29.9%	0.184
DCR	94.2%	79.1%	0.009

Abbreviations: AE, adverse event; CR, complete response; DCR; disease control rate; ORR, overall response rate; PD, progressive disease; PR, partial response; SD, stable disease.

The radiological best response rates stratified by the presence or absence of AEs that were not significantly related to progression‐free survival by multivariate analysis are listed in Table 7 (immune related) and Table 8 (non‐immune related). The radiological best response rates and DCR were significantly different when stratified by the presence or absence of treatment‐related proteinuria of any grade (*p* = 0.042 and 0.001) (Table 8). The radiological best response rates were significantly different when stratified by the presence or absence of treatment‐related decreased appetite of any grade (*p* = 0.042) (Table 8).

**TABLE 7:** Therapeutic response by immune‐related AEs

	Immune‐related liver injury			Immune‐related endocrine dysfunction		
	Yes (*n* = 30)	No (*n* = 233)	*p* value	Yes (*n* = 29)	No (*n* = 234)	*p* value
CR	1 (3.3%)	12 (5.2%)	0.901	1 (3.4%)	12 (5.1%)	0.265
PR	10 (33.3%)	61 (26.2%)		11 (37.9%)	60 (25.6%)	
SD	14 (46.7%)	118 (50.6%)		15 (51.7%)	117 (50.0%)	
PD	5 (16.7%)	42 (18.0%)		2 (6.9%)	45 (19.2%)	
ORR	36.7%	31.3%	0.540	41.4%	30.8%	0.292
DCR	83.3%	82.0%	1.000	93.1%	80.8%	0.126

Abbreviations: AE, adverse event; CR, complete response; DCR; disease control rate; ORR, overall response rate; PD, progressive disease; PR, partial response; SD, stable disease.

**TABLE 8:** Therapeutic response by non–immune‐related AEs

	Proteinuria			Fatigue			Decreased appetite		
	Yes (*n* = 87)	No (*n* = 176)	*p* value	Yes (*n* = 72)	No (*n* = 191)	*p* value	Yes (*n* = 61)	No (*n* = 202)	*p* value
CR	5 (5.7%)	8 (4.5%)	0.044	1 (1.4%)	12 (6.3%)	0.218	0 (0.0%)	13 (6.4%)	0.042
PR	29 (33.3%)	42 (23.9%)		24 (33.3%)	47 (24.6%)		21 (34.4%)	50 (24.8%)	
SD	45 (51.7%)	87 (49.4%)		33 (45.8%)	99 (51.8%)		26 (42.6%)	106 (52.5%)	
PD	8 (9.2%)	39 (22.2%)		14 (19.4%)	33 (17.3%)		14 (23.0%)	33 (16.3%)	
ORR	39.1%	28.4%	0.092	34.7%	30.9%	0.556	34.4%	31.2%	0.641
DCR	90.8%	77.8%	0.010	80.6%	82.7%	0.719	77.0%	83.7%	0.255

Abbreviations: AE, adverse event; CR, complete response; DCR; disease control rate; ORR, overall response rate; PD, progressive disease; PR, partial response; SD, stable disease.

## 4 DISCUSSION

In this multicenter investigation, multivariate analysis for survival showed correlations between AEs and outcomes. Specifically, treatment‐related hypertension of any grade was independently related to good progression‐free survival in patients with HCC who were treated with Atez/Bev, the combination of an immune checkpoint inhibitor (Atez) and a molecular targeted agent (Bev) that was developed as 1st‐line systemic therapy for patients with advanced unresectable HCC. In addition, treatment‐related fatigue of any grade was independently related to poor overall survival. Furthermore, in the evaluation of therapeutic response, the DCR was significantly higher in patients who developed treatment‐related hypertension of any grade than in those who did not. These results of this study suggest that treatment‐related hypertension is related to good therapeutic response and progression‐free survival, whereas fatigue is related to poor outcomes in patients with advanced unresectable HCC who were treated with Atez/Bev.

Regarding safety in the phase 3 IMbrave150 study,^3^ grade ≥ 3 treatment‐related AEs were higher, but not significantly so, in the sorafenib patient group (46%, 71/156) than in the Atez/Bev patient group (36%, 117/329). Among AEs with an incidence ≥10%, those that were common in the sorafenib patient group, such as hand–foot skin reaction, diarrhea, hypertension, and decreased appetite, were few in the Atez/Bev patient group. Immune‐related liver injury and proteinuria were a little more than usual in the Atez/Bev patient group than in the sorafenib patient group; however, these events were grade ≤ 2. In the present study, three treatment‐related AEs, namely decreased appetite of any grade, proteinuria of any grade, and fatigue of any grade, occurred with an incidence of ≥20% in patients with HCC who were treated with Atez/Bev as 1st‐line systemic therapy. Regarding immune‐related AEs, liver injury of any grade and endocrine dysfunction of any grade were observed in approximately 10% of this cohort. The current study showed an increase in the incidence of major AEs compared to the first report of our multicenter study,^14^ but the rates of these AEs incidences were almost the same as in our most recent reports.^15^, ^16^, ^17^, ^18^, ^19^, ^20^, ^21^

Studies of several malignancies, such as lung cancer and malignant melanoma, reported that patients treated with immune checkpoint inhibitors who developed immune‐related AEs showed significantly better prognosis than those who did not develop these AEs.^5^, ^6^, ^7^, ^8^ However, this relationship was not adequately investigated in patients with HCC who were treated with immune checkpoint inhibitors. In this study, multivariate analysis demonstrated that immune‐related AEs did not affect outcomes in patients with HCC who were treated with Atez/Bev.

Some tyrosine kinase inhibitor–related AEs have been shown to correlate with good HCC patient outcomes; for instance, sorafenib‐, regorafenib‐, and lenvatinib‐related dermatological AEs were associated with improved survival.^11^, ^12^, ^13^ Tyrosine kinase inhibitor–related diarrhea and hypertension have also related to good outcomes in patients with unresectable HCC.^11^ Rapposelli et al.^13^ conducted a study of patients with unresectable HCC who were treated with lenvatinib and reported that the development of treatment‐related hypertension of grade ≥ 2 independently predicted good overall survival (HR, 0.66; 95% CI, 0.46–0.93, *p* = 0.019) in multivariate analysis. The development of hand–foot skin reaction, hypertension, and diarrhea are all likely to be associated with the mechanism of action of tyrosine kinase inhibitors. These drugs may cause hypertension by suppressing vascular endothelial growth factor (VEGF)‐mediated upregulation of nitric oxide synthase, thereby inhibiting vasomotor function and promoting degeneration of small blood vessels.^32^

Bev is a humanized monoclonal antibody that connects to all isoforms of VEGF.^33^ By connecting to VEGF and preventing it from interaction with its receptors, Bev inhibits angiogenesis and induces the regression of newly formed microvessels and “normalization” of abnormal tumor vascularization.^34^ In addition, VEGF inhibition is directly associated with the onset of hypertension, a recognized class effect of anti‐angiogenic treatments, such as Bev. As VEGF is required to preserve the normal endothelial cell function and homeostasis of vascular,^35^, ^36^ stopping the pathway of VEGF can lead to hypertension and endothelial dysfunction. The mechanism of Bev‐related hypertension is not sufficiently clarified, however several hypotheses have been reported.^37^, ^38^, ^39^ As with tyrosine kinase inhibitors, it is speculated that one important factor is the decreased production of nitric oxide that appears when VEGF is inhibited. Several reports showed that Bev‐related hypertension was correlated with favorable outcomes in patients with malignancies such as breast cancer, lung cancer, colorectal cancer, and renal cell carcinoma.^10^, ^40^, ^41^, ^42^ Although the mechanism of the relationship between Bev‐related hypertension and treatment responsiveness is not fully understood, treatment‐related hypertension may be a phenotypic clinical marker of a specific genotype associated with a better response to Bev.^43^ In this setting, titrating Bev doses may be less effective because patients who are less prone to treatment‐related hypertension may have tumor genotypes that are originally resistant to this drug, despite adequate drug concentrations. Certain genotypes have been related to both the therapeutic response to Bev and the onset of Bev‐related hypertension. In this study, we clarified that hypertension related to Bev therapy was correlated with good outcomes in patients with HCC who were treated with Atez/Bev. Therefore, it is considered important that patients with HCC who are receiving Atez/Bev continue the appropriate use of antihypertensive medications when they develop treatment‐related hypertension.

Our finding that treatment‐related fatigue was associated with worse outcomes is consistent with another report showing that this AE was negative prognostic factor in patients with HCC treated with systematic therapy.^44^ However, significantly more patients with treatment‐related fatigue had advanced BCLC stage, which may have affected overall survival. In a 9‐week landmark analysis, we recently demonstrated that Bev interruption due to AEs was associated with therapeutic efficacy.^16^ In our reports,^16^ we clarified that Bev interruption was significantly correlated with both progression‐free survival (HR, 1.75; 95% CI, 1.09–2.82; *p* = 0.021) and overall survival (HR, 2.55; 95% CI, 1.28–5.07; *p* = 0.008). In addition, we showed that AEs such as liver injury (including immune related), decreased appetite, and proteinuria occurred more frequently in patients with Bev interruption than in those without it. In this study, decreased appetite and proteinuria were not related to progression‐free or overall survival. They are probably due to the fact that our previous study^16^ used a landmark analysis and may have been influenced by differences in the consideration of the time of development of AEs. In this study, no patient had treatment‐related hypertension of grade 4, and hypertension as an AE was considered to have little association with Bev interruption.

The strength of this study was that it determined the relationship between treatment‐related AEs and outcomes in patients treated with Atez/Bev, the current first‐line systematic therapy for unresectable HCC, in a first‐line setting.

This study is not without limitations, such as the hospital‐based population and retrospective design. Although the study included patients who were treated with Atez/Bev in a 1st‐line setting at multiple centers in Japan, future prospective studies should include a larger number of patients and those recruited on a nationwide basis, and should also use a longer‐term follow‐up period. This study did not analyze the treatment of HCC after Atez/Bev therapy. As sequential systemic treatment may influence prognosis, especially overall survival, further studies that include an assessment of HCC treatment after Atez/Bev therapy are also warranted. There was concern that grouping patients after the start of observation may introduce immortality time bias. Specific statistical methods, such as the landmark method of grouping by the onset of each AE, should be considered in the future. The current study did not sufficiently investigate the timing of the onset of each AE, and detailed studies of the relationship between the onset of each AE and its grade are needed in the future.

In conclusion, treatment‐related hypertension was related to good outcomes in HCC patients treated with Atez/Bev. Conversely, treatment‐related fatigue was related to poor outcome in these patients. Therefore, careful management of AEs is necessary to maintain patients' quality of life, minimize the need for treatment discontinuation, and achieve desirable outcomes such as prolonged survival. More studies are needed to confirm these findings in other patient populations.

## AUTHOR CONTRIBUTIONS

**Toshifumi Tada:** Conceptualization (lead); data curation (equal); formal analysis (lead); methodology (lead); writing – original draft (lead); writing – review and editing (lead). **Takashi Kumada:** Conceptualization (equal); formal analysis (supporting); investigation (supporting); methodology (supporting); project administration (lead); supervision (lead); writing – original draft (supporting); writing – review and editing (supporting). **Atsushi Hiraoka:** Conceptualization (equal); data curation (lead); methodology (equal); project administration (lead); supervision (supporting); writing – original draft (supporting); writing – review and editing (supporting). **Masashi Hirooka:** Data curation (equal). **Kazuya Kariyama:** Data curation (equal); writing – original draft (supporting); writing – review and editing (supporting). **Joji Tani:** Data curation (equal). **Masanori Atsukawa:** Data curation (equal). **Koichi Takaguchi:** Data curation (equal). **Ei Itobayashi:** Data curation (equal). **Shinya Fukunishi:** Data curation (equal). **Kunihiko Tsuji:** Data curation (equal). **Toru Ishikawa:** Data curation (equal). **Kazuto Tajiri:** Data curation (equal). **Hironori Ochi:** Data curation (equal). **Satoshi Yasuda:** Data curation (equal). **Hidenori Toyoda:** Data curation (equal). **Chikara Ogawa:** Data curation (equal). **Takashi Nishimura:** Data curation (equal). **Takeshi Hatanaka:** Conceptualization (equal); data curation (equal); formal analysis (supporting); methodology (equal); project administration (equal); writing – original draft (supporting); writing – review and editing (supporting). **Kakizaki Satoru:** Data curation (equal). **Noritomo Shimada:** Data curation (equal). **Kazuhito Kawata:** Data curation (equal). **Fujimasa Tada:** Data curation (equal). **Hideko Ohama:** Data curation (equal). **Kazuhiro Nouso:** Data curation (equal). **Asahiro Morishita:** Data curation (equal). **Akemi Tsutsui:** Data curation (equal). **Takuya Nagano:** Data curation (equal). **Norio Itokawa:** Data curation (equal). **Tomomi Okubo:** Data curation (equal). **Taeang Arai:** Data curation (equal). **Michitaka Imai:** Data curation (equal). **Hisashi Kosaka:** Data curation (equal). **Atsushi Naganuma:** Data curation (equal). **Yohei Koizumi:** Data curation (equal). **Shinichiro Nakamura:** Data curation (equal). **Masaki Kaibori:** Data curation (equal). **Hiroko Iijima:** Data curation (equal); funding acquisition (lead); supervision (supporting). **Yoichi Hiasa:** Data curation (equal); supervision (supporting).

## FUNDING INFORMATION

This work was supported by JSPS KAKENHI 21 K07902.

## CONFLICT OF INTEREST

Toshifumi Tada (tadat0627@gmail.com): lecture fees from AbbVie and Eisai. Atsushi Hiraoka (hirage@m.ehime-u.ac.jp): lecture fees from Bayer, Eisai, Eli Lilly, and Otsuka. Takashi Kumada (takashi.kumada@gmail.com): lecture fees from Eisai. Hidenori Toyoda (hmtoyoda@spice.ocn.ne.jp): lecture fees from AbbVie, Bayer, Eisai, Gilead, and Terumo. Takeshi Hatanaka (hatanaka@qk9.so-net.ne.jp): lecture fees from Eisai. None of the other authors have potential conflicts of interest to declare.

## ETHICS APPROVAL AND PATIENT CONSENT

Written informed consent was obtained from each patient before study enrollment. The study protocol conformed to the ethical guidelines of the Declaration of Helsinki. The study was approved by the institutional ethics review committee of Ehime Prefectural Central Hospital (IRB No. 30–66) (UMIN000043219).

## Supporting information


Table S1.

## Data Availability

The datasets are available from the corresponding author on reasonable request.
