# Supplementary material for: Adverse events as potential predictive factors of therapeutic activity in patients with unresectable hepatocellular carcinoma treated with atezolizumab plus bevacizumab
Source: Cancer Med. 2022 Dec 14;12(7):7772–83. doi: 10.1002/cam4.5535 (PMC10134356; doi:10.1002/cam4.5535)
Supplement: Supplementary file 1 — Table S1. [file CAM4-12-7772-s001.docx]

**Supplementary table 1. Patient characteristics stratified by the presence or absence of treatment-related fatigue of any grade**

|  | **Treatment-related fatigue of any grade** | | **p value** |
| --- | --- | --- | --- |
|  | **Yes (n=72)** | **No (n=191)** |  |
| Age* (years) | 73.5 (68.0–79.3) | 74.0 (67.0–79.0) | 0.957 |
| Sex (female/male) | 16/56 | 39/152 | 0.737 |
| ECOG-PS (0/1/≥2) | 54/13/5 | 162/22/7 | 0.156 |
| Body mass index (kg/m^2^) | 23.8 (20.7–25.2) | 23.7 (21.6–26.6) | 0.300 |
| Etiology of HCC (hepatitis B/C/B+C/non-B, non-C) | 12/23/0/37 | 29/71/1/90 | 0.816 |
| Albumin (g/dL)* | 3.7 (3.3–4.1) | 3.8 (3.4–4.1) | 0.269 |
| Total bilirubin (mg/dL)* | 0.8 (0.6–1.1) | 0.8 (0.6–1.1) | 0.521 |
| Platelet count (×10^3^/m^3^) * | 13.9 (9.8–20.5) | 13.7 (10.–19.3) | 0.841 |
| Prothrombin time (%)* | 88 (77–97) | 89 (81–98) | 0.895 |
| α-fetoprotein level (ng/mL)* | 42.5 (7.0–193.9) | 30.7 (5.9–392.6) | 0.693 |
| Child–Pugh score (5/6/≥7) | 44/21/7 | 119/59/13 | 0.705 |
| BCLC stage (≤A/B/≥C) | 1/30/41 | 20/70/101 | 0.037 |
| Follow-up duration* (months) | 7.7 (4.6–11.1) | 8.5 (5.5–12.2) | 0.263 |

*Data expressed as medians (interquartile range).

ECOG-PS, Eastern Cooperative Oncology Group Performance Status; HCC, hepatocellular carcinoma; BCLC, Barcelona Clinic Liver Cancer.
